# Tolérance de l’évérolimus en pratique clinique: étude retrospective

**DOI:** 10.11604/pamj.2020.36.26.16580

**Published:** 2020-05-21

**Authors:** Leila Afani, Rhizlane Belbaraka, Ahmad Awada

**Affiliations:** 1 Department of Medical Oncology, CHU Mohamed VI, Marrakech, Morrocco; 2 Oncology Department, Jules Bordet Institute, Brussels, Belgium

**Keywords:** Evérolimus, tolérance, cancer du rein, cancer du sein, Everolimus, tolerance, kidney cancer, breast cancer

## Abstract

L’évérolimus est un inhibiteur mTOR ayant démontré une activité clinique dans plusieurs tumeurs solides notamment dans le cancer du rein après une première ligne à base d’un TKI anti VEGF et dans le cancer du sein en association avec l’exemesthane après échec d’un antiaroamatase. L’objectif de ce travail est d’analyser le profil de tolérance de l’évérolimus dans le cancer du sein et le cancer du rein en pratique clinique. Il s’agit d’une étude rétrospective portant sur les patients suivis pour cancer du sein et cancer du rein durant la période s’étendant de janvier 2008 à janvier 2015. Tous les patients ont reçu l’évérolimus à la dose quotidienne de 10 mg seul ou en association avec l’exemesthane pour le cancer du sein. Les effets indésirables ont été gradés selon la classification du *National Cancer Institute Common Terminology Criteria for Adverses version 4.0 (NCI-CTCAE)*. Au total 100 patients ont été inclus: 76 patientes avec cancer du sein et 24 patients avec cancer rénal. La durée médiane de traitement était estimée à 5,7 mois. Le traitement a été arrêté dans plus de 70% des cas pour intolérance. Les principaux effets indésirables avec une incidence de plus de 30% pour tous les grades étaient la mucite, le rash, la fatigue, l’anémie, la lymphopénie, l’hyperglycémie, l’hyperlipidémie et les infections. Les toxicités grade 3-4 avec incidence élevée étaient la mucite, la pneumopathie non infectieuse et les infections. Le taux d’arrêt du traitement pour intolérance reste élevé en comparaison avec les données de la literature. Une attention particulière doit être accordée à la mucite, l’effet immunosuppresseur de traitement et la pneumopathie non infectieuse et ce dès le début du traitement.

## Abstract (en)

Everolimus is a mTOR inhibitor which demonstrates clinical activity in several solid tumors, especially in kidney cancer after first line TKI anti VEGF treatment and in breast cancer in association with exemesthane after failure of aroamatase inhibitors. The purpose of this study was to analyze in clinical practice the tolerance of everolimus in patients with breast cancer and kidney cancer. We conducted a retrospective study on patients followed up for breast cancer and kidney cancer over the period January 2008 - January 2015. All patients received everolimus at a dosage of 10 mg/day alone or in association with exemesthane for breast cancer. Adverse reactions were classified according to the National Cancer Institute Common Terminology Criteria for Adverses version 4.0 (NCI-CTCAE). A total of 100 patients were enrolled in the study: 76 patients with breast cancer and 24 patients with kidney cancer. The median follow-up period was 5.7 months. Treatment was stopped in more than 70% of the cases because of intolerance. The main adverse events, with a prevalence of more than 30% for all grades were mucositis, rash, fatigue, anemia, lymphopenia, hyperglycaemia, hyperlipidemia and infections. Mucositis, noninfectious pneumopathies and infections had high incidence of toxicity grade 3-4. Treatment discontinuation rate due to intolerance is high compared to literature data. At the beginning of treatment, particular attention should be given to mucositis, the immunosuppressive effect of treatment and non-infectious pneumopathies.

## Introduction

L’évérolimus est un analogue de la rapamycine avec des propriétés immunosuppressives et antiprolifératives. Il est administré par voie orale à la dose quotidienne de 10 mg. L’évérolimus est indiqué dans le traitement de plusieurs tumeurs ; le cancer du rein avancé après progression sous sunitinib ou sorafenib, les tumeurs neuroendocrines du pancréas localement avancées ou métastatiques, l’astrocytome sous épendymaire géant et l’angiomyolipome rénal dans le cadre de la sclérose tubéreuse de Bourneville. Plus récemment, l’évérolimus a été approuvé dans le cancer du sein avancé RH+ HER2- chez les femmes ménopausées après échec d’un traitement par letrozole ou anastrozole [1]. Les principaux effets indésirables liés à l’évérolimus observés par ordre de fréquence sont la stomatite qui est caractérisée par une composante inflammatoire mimant une stomatite aphteuse [2]. Le rash avec des lésions maculopapuleuse ou papulo-pustuleuses associé souvent à un prurit [3]. La pneumopathie non infectieuse infiltrant le poumon pouvant être liée à une médiation immunologique. Les symptômes respiratoires incluent la toux, la dyspnée, l’hypoxie et plus rarement l’épanchement pleural. Elle peut être asymptomatique. Au niveau radiologique, il s’agit souvent d’opacités en verre dépoli prédominant dans les lobes inférieurs [4]. Les infections sont un effet indésirable souvent rapporté avec l’évérolimus. Elles sont dues aux propriétés immunosuppressives de l’évérolimus prédisposant aux infections bactériennes, virales ou fongiques [5]. Des perturbations métaboliques tels que l’hyperglycémie et l’hyperlipidémie sont souvent observées avec l’évérolimus [6]. Toutes les anomalies hématologiques tels que l’anémie, la thrombopénie, la lymphopénie et les neutropénies peuvent survenir mais avec une incidence moins élevée [7]. L’objectif de ce travail est d’évaluer l’incidence des effets indésirables liés à l’évérolimus en pratique clinique et d’analyser le profil de tolérance en fonction du type de tumeur.

## Méthodes

Il s’agit d’une étude rétrospective portant sur 100 patients ayant été traités à l’institut Jules Bordet depuis janvier 2008 à janvier 2015. Toutes les données ont été collectées via les dossiers informatiques Oribase de l’institut Jules Bordet. Tous les patients ont reçu l’évérolimus à la dose de 10 mg par jour seul pour les tumeurs du rein et en association au letrozole pour les tumeurs du sein. Les effets indésirables ont été gradés selon la classification du National Cancer Institute Common Terminology Criteria for Adverses version 4.0 (NCI-CTCAE). La base des données a été construite au moyen du logiciel Microsoft Access 2007. Les analyses statistiques sont essentiellement descriptives. Les grades des effets indésirables I/II/III/IV ont été rapportés en fonction de la période de traitement (les premiers trois mois, entre 3 et 6 mois et au-delà de 6 mois). Le grade le plus bas durant chaque période a été noté. Pour les effets indésirables avec un taux d’incidence dépassant 10%, l’intervalle de confiance 95% a été calculé. Toutes les analyses statistiques ont été réalisées avec le logiciel SPSS.

## Résultats

De 2008 à 2015, 100 patients ont reçu un traitement à base d’évérolimus. Les caractéristiques générales des patients sont illustrées dans le Tableau 1. L’âge médian était de 59ans dans le cancer du sein et 62 ans pour le groupe du cancer du rein. 59 patients avaient un index de performans status de 1. Sur les 100 patients, 29 patients avaient des comorbidités (diabète, dyslipidémie, hypertension artérielle et hypothyroidie). Le type histologique dominant était le carcinome canalaire infiltrant dans le cancer du sein (78%) et le carcinome à cellules claires (92%) dans le cancer du rein. La durée médiane de traitement était estimée à 5.7 mois (0.9 à 63.2) dans le cancer du sein et de 5.5 mois (0.9 à 71,1) dans le cancer du rein. Le traitement a été arrêté chez 89 patients. Pour les patients suivis pour cancer du sein, 52 patients ont arrêté le traitement pour intolérance et 15 patients suite à une progression. Pour le cancer du rein, 16 patients ont arrêté le traitement pour intolérance (73%) et 6 patients suite à une progression (27%). 11 patients prenaient toujours leur traitement au moment de la collecte des données. Les principaux effets indésirables ont été rapportés dans le Tableau 2. Dans le cancer du sein, la pneumopathie interstitielle de grade 3-4 a été observée chez deux patients. Sur le plan digestif, l’incidence des mucites était élevée au début de traitement avec un grade 3-4 observé chez 14 patients (18%). Au niveau hématologique, on a noté un seul grade 3-4 de l’anémie et la thrombopénie. Par contre 3 cas ont présenté un grade 3-4 de lymphopénie. Sur le plan métabolique, la toxicité la plus fréquente était l’hypercholestérolémie qui a été observée chez 54 patients (71%) suivie de l’hyperglycémie (42%). Le taux d’incidence de grade 3-4 d’infections était de 9% aux premiers six mois. La fatigue était observée chez 59 patients (78%) en début de traitement. L’apparition de rash était observée chez 28 patients en début de traitement. Dans le cancer du rein, 4 cas de pneumopathie interstitielle de grade 3-4 ont été objectivé au début de traitement. La tolérance digestive était marquée par un taux élevé de mucite (71%) avec 4 cas de toxicité grade 3-4. Au niveau hématologique, l’anémie et la lymphopénie ont été rapporté chez 14 patients avec un seul cas de grade 3-4. Au niveau métabolique, l’hypercholestérolémie et l’hypertriglycéridémie étaient observées chez 18 et 15 patients. L’hyperglycémie grade 3-4 a été rapportée chez 5 patients. Le taux d’infections grade 3-4 était de 20%. Un seul cas de fatigue grade 3-4 a été mentionné. L’apparition de rash et d’œdèmes est observé chez 7 et 6 patients.

**Tableau 1:** Caractéristiques générales des patients

Caractéristiques	Cancer du sein N= 76	Cancer du rein N= 24
**Age médian**	59 (39 à 82ans)	62 (53 à 84ans)
**Sexe**		
Masculin	0 (0%)	17 (71%)
Feminin	76 (100%)	7 (29%)
**Types de comorbidités**		
Diabète	7 (9%)	4 (17%)
Dyslipidémie	5 (7%)	5 (21%)
Hypertension artérielle	15 (20%)	6 (25%)
Hypothyroidie	0 (0%)	1 (4%)
**Type histologique**		
Carcinome canalaire infiltrant	59 (78%)	-
Carcinome lobulaire infiltrant	17 (22%)	-
Carcinome rénal à cellules claires	-	22 (92%)
Carcinome rénal non ç cellules claires	-	2 (8%)
**Nombre de sites métastatiques**		
1	40 (53%)	6 (25%)
>2	36 (47%)	18 (75%)
**OMS avant évérolimus**		
0	18 (20%)	2 (8%)
1	44 (59%	15 (63%)
2	14 (21%)	7 (29%)

**Tableau 2:** Incidence des effets indésirables tous grades confondus et les grades 3-4 dans le cancer du sein et le cancer du rein

Principaux effets indésirables	Cancer du sein N=76 (%)		Cancer du rein N=24(%)	
	Tous grades	Grades 3-4	Tous grades	Grades 3-4
**Digestifs:**				
Stomatite	56(74)	14(18)	17(71)	4(17)
Nausée	4(5)	0(0)	5(21)	1(4)
Diarrhée	12(16)	1(1)	5(21)	1(4)
**Respiratoires:**				0
Dyspnée	17(22)	4(5)	7(29)	2(8)
Pneumonitis	7(9)	2(3)	6(25)	4(17)
**Autres :**				
Rash	28(37)	0(0)	7(29)	0(0)
Fatigue	59(78)	3(4)	21(88)	1(4)
Infections	59(78)	7(9)	8(33)	2(8)
Oedèmes	12(16)	0(0)	6(25)	0(0)
**Hématologiques:**				
Anémie	38(50)	1(1)	14(58)	1(4)
Lymphopénie	36(47)	3(4)	14(58)	1(4)
Thrombopénie	13(17)	1(1)	1(4)	0(0)
**Métaboliques:**				
Hyperglycémie	32(42)	4(5)	14(58)	2(8)
Hypercholestérolémie	54(71)	0(0)	18(75)	0(0)
Hypertriglycéridémie	26(34)	1(1)	15(63)	0(0)

## Discussion

Plusieurs études ont testé la tolérance et l’efficacité clinique de l’évérolimus associé à l’hormonothérapie dans le cancer du sein. L’évérolimus a été testé initialement en association avec le letrozole en situation métastatique. Les effets indésirables rapportés étaient la mucite (50%), la fatigue (44.4%), la diarrhée (38.9%), le rash (33.3%). 4 patientes ont arrêté le traitement pour cause de toxicité [8]. En néoadjuvant, le taux de mucite était de (36.5%), le rash (20.4%), la fatigue (12.4%), l’hyperglycémie (13.1%) et l’hypercholestérolémie (16.1%). Les toxicités grade 3-4 étaient observées dans 22.6%[9]. L’éverolimus a été testé dans une phase II en association avec du tamoxifène dans le cancer du sein métastatique résistant aux inhibiteurs de l'aromatase. La mucite grade 3-4 a été observé dans 11% des cas. Et 20% de patientes ont nécessité des réductions de dose suite à la toxicité [10]. Dans l’étude Bolero 2, les toxicités tous grades confondus avec une incidence >30% étaient la mucite, le rash et la fatigue. Le même taux d’incidence était observé dans notre série et s’y ajoute d’autres effets indésirables; l’anémie, la lymphopénie, l’hyperglycémie et l’hypercholestérolémie. Les toxicités grade 3-4 les plus fréquentes avec une incidence égale ou plus de 3% étaient la mucite, la fatigue, la dyspnée, la pneumopathie, l’anémie, la thrombopénie et l’hyperglycémie dans l’étude Boléro [11]. Dans notre série s’ajoutent les infections, la lymphopénie et la neutropénie. Concernant le cancer du rein, dans la première phase II testant l’évérolimus, la plupart des effets indésirables étaient de grade 1-2. Les toxicités de grade 3 étaient la pneumopathie non infectieuse (n=7, 18%), l’hyperglycémie et la thrombopénie (n=3, 8%). Aucun grade 4 n’a été mentionné [12,13]. Dans l’étude Phase III Record -1, les effets indésirables les plus fréquents (incidence de plus de 30%) tous grades confondus étaient la stomatite (44%), les infections (37%) et la fatigue (31%). Les toxicités grade 3-4 avec une incidence dépassant 3% étaient la mucite (4%) les infections (7%), la dyspnée (6%), la fatigue (5%), la pneumopathie non infectieuse (4%). Concernant les anomalies de laboratoires classées grade 3-4, l’anémie (12%), la lymphopénie (16%) et l’hyperglycémie (15%) [14]. Ces données rejoignent exactement ceux retrouvées dans notre série. En comparant la tolérance de l’évérolimus dans le cancer du sein et le cancer du rein, l’incidence des principaux effets indésirables dans les deux phases III testant l’évérolimus dans le cancer du sein et le cancer du rein ainsi que dans notre série restent comparables (Tableau 3). La durée médiane de traitement dans notre série était de 22.8 semaines dans le cancer du sein et de 22 semaines dans le cancer du rein. Dans la littérature, elle était de 23.9 semaines dans le cancer du sein et de 20.1 semaines dans le cancer du rein [2,14]. Cependant le taux d’arrêt de l’évérolimus et les réductions de doses étaient plus élevés dans le cancer du sein. Dans l’étude Bolero, il y’avait 26% de taux d’arrêt de traitement à cause des effets indésirables contre 13% dans l’étude RECORD-1. Cette différence s’observe aussi bien dans notre série. Cela pourrait s’expliquer par la nouveauté de l’indication de l’évérolimus dans le cancer du sein. Un grand nombre de patients sont lourdement prétraités avant de recevoir l’évérolimus. Un autre argument serait que les praticiens ne sont pas habitués à la gestion des effets indésirables de cette nouvelle molécule, d’une part et la présence de plusieurs options thérapeutiques disponibles dans le cancer du sein d’autre part. Un autre point serait que la fréquence du cancer du sein métastatique type luminal est élevé par rapport au cancer du rein. Ainsi en pratique clinique on met de plus en plus les malades de cancer du sein sous évérolimus. Les données de notre série confirment cette fréquence avec seulement 24 patients suivis pour cancer du rein, traités par évérolimus sur une durée de 7ans. La plupart des effets indésirables surviennent le premier trimestre du début du traitement. Ainsi la détection précoce des toxicités et la réduction des doses permettraient de diminuer l’incidence de la gravité des toxicités liées à l’évérolimus [15-16].

**Tableau 3:** Incidence comparative des effets indésirables dans le cancer du sein et le cancer du rein

Effets indésirables	Sein métastatique Etude Bolero (n= 482) %		Rein métastatique Etude Record-1 (n= 274) %		Notre série Cancer du sein (n=76)%		Cancer du rein Notre série (n=24)%	
	Tous grades	Grade 3-4	Tous grades	Grade 3-4	Tous grades	Grades 3-4	Tous grades	Grades 3-4
**Stomatite**	67	8	44	4	74	18	71	17
**Rash**	39	1	29	1	37	0	29	0
**Fatigue**	36	4	31	5	78	4	88	4
**Infections**	50	5	37	10	21	9	33	8
**Pneumopathie non infectieuse**	19	4	14	4	9	3	25	17
**Dyspnée**	21	4	24	7	22	5	29	8
**Anémie**	68	6	92	13	50	1	58	4
**Lymphopénie**	54	11	51	18	47	4	58	4
**Thrombopénie**	31	2	23	1	17	1	4	0
**Hyperglycémie**	69	9	57	15	42	5	58	8
**Hypercholestérolémie**	70	0	77	4	71	0	75	0
**Hypertriglycéridémie**	50	0	73	<1	34	1	63	0

## Conclusion

En pratique clinique, le taux d’arrêt du traitement pour intolérance est assez élevé en comparaison avec les données de la literature. Une attention particulière doit être accordée à la mucite, l’effet immunosuppresseur du traitement et la pneumopathie non infectieuse et ce dès le début du traitement. Les toxicités liées au traitement restent cependant acceptables. La plupart des effets indésirables surviennent très tôt. D’où la nécessité de bien les expliquer aux patients dès le début du traitement. Le diagnostic rapide de ces effets indésirables permet une prise en charge précoce et adéquate ainsi une meilleure compliance au traitement.

### État des connaissances actuelles sur le sujet

- L’évérolimus est un traitement indiqué dans le cancer du sein et le cancer du rein;
- Les principaux effets indésirables de l’évérolimus sont le rash, la mucite, la pneumpoathie non infectieuse, les infections et les perturbations métaboliques;
- L’incidence des effets indésirables dans le cancer du sein et le cancer du rein sont comparables.

### Contribution de notre étude à la connaissance

- Le taux d’arrêt du traitement pour intolérance au traitement était élevé dans le cancer du sein;
- L’évérolimus parait mieux toléré dans le traitement du cancer du rein;
- La plupart des effets indésirables survenaient au premier trimestre.

## Conflits d’intérêts

Les auteurs ne déclarent aucun conflit d'intérêts.
